# Meditation for perioperative pain and anxiety: A systematic review

**DOI:** 10.1002/brb3.3640

**Published:** 2024-07-29

**Authors:** Rami Rajjoub, Sally El Sammak, Tamim Rajjo, Noora S. Rajjoub, Bashar Hasan, Samer Saadi, Adel Kanaan, Mohamad Bydon

**Affiliations:** ^1^ Department of Neurologic Surgery Mayo Clinic Rochester Minnesota USA; ^2^ Mayo Clinic Neuro‐Informatics Laboratory Mayo Clinic Rochester Minnesota USA; ^3^ Department of Family Medicine Mayo Clinic Rochester Minnesota USA; ^4^ Robert D. and Patricia E. Kern Center for the Science of Health Care Delivery Mayo Clinic Rochester Minnesota USA; ^5^ Evidence‐Based Practice Center Mayo Clinic Rochester Minnesota USA

**Keywords:** anxiety, meditation, pain, perioperative outcomes, review

## Abstract

**Introduction:**

Effective pain and anxiety management during the perioperative phase remains a challenge for patients undergoing surgeries and other invasive procedures. The current standard of care involves prescribing analgesics to treat these conditions; however, there has been recent interest in applying multimodal strategies that limit the use of these medications. One such modality is meditation, which has been shown to be effective in alleviating various physical and psychological symptoms in other settings. This systematic review aims to assess how current meditative practices affect perioperative pain and anxiety.

**Methods:**

We conducted a systematic review of randomized controlled trials following the Preferred Reporting Items for Systematic Reviews and Meta‐Analyses guidelines. A comprehensive literature search was conducted using PubMed MEDLINE, Embase, PsycINFO, APA PsycINFO, EBM Reviews, Scopus, and Web of Science for all available dates. Our primary outcomes of interest were patient‐reported pain and anxiety scores using the Visual Analog Scale, the Brief Pain Inventory, the Depression Anxiety Stress Scale, the State‐Trait Anxiety Inventory (STAI), and the Hospital Anxiety and Depression Scale (HADS). For the HADS and STAI scales, only the anxiety and anxiety‐state subgroups were reported, respectively.

**Results:**

The literature search yielded 1746 articles. A total of 286 full‐text articles were screened, and 16 studies were included in this systematic review. A total of eight studies assessed pain scores after invasive procedures; five reported improvements in pain scores, and three reported no change after meditative practices. Ten studies assessed anxiety outcomes after invasive procedures: nine reported a decrease in overall anxiety levels as a result of meditation practices while one study reported no change in anxiety scores.

**Conclusion:**

Data from this limited literature suggests that different meditation practices could be effective in alleviating pain and anxiety within the perioperative phase for patients undergoing various types of invasive procedures. Future prospective studies are needed to determine whether routine meditation in the perioperative setting is effective in mitigating perioperative pain and anxiety.

## Graphical abstract

This systematic review assesses the impact of meditation on perioperative pain and anxiety management. The analysis included 16 randomized controlled trials, finding that meditation practices generally reduced anxiety and, in some cases, alleviated pain in patients undergoing invasive procedures. While the results suggest potential benefits, the study highlights the need for more rigorous research to confirm the efficacy of meditation in perioperative settings.

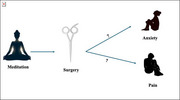

## 1 INTRODUCTION

Preoperative and perioperative pain and anxiety have been associated with negative postoperative consequences such as depression, sleep disturbances, neurocognitive degeneration, and overall worse physical outcomes in both short‐ and long‐term follow‐ups (Ayers et al., 2004; Badura‐Brzoza et al., 2009; Caracciolo & Giaquinto, 2005; Dowsey et al., 2014). The current standard of care for perioperative pain control involves administering analgesics, most often opioids (Shanthanna et al., 2021). However, the addictive nature of these analgesics, along with the impaired rehabilitation process, prolonged duration of stay, and complications from oversedation has led physicians to seek alternate options (Argoff, 2014; Haisley et al., 2020). Moreover, increased levels of stress and fear of upcoming invasive procedures have led to preoperative anxiety rates of up to 80% among patients across various fields (Guerrier et al., 2021; Gürsoy et al., 2016; Theunissen et al., 2018). Some studies have even demonstrated that the extended stress associated with prolonged preoperative anxiety may lead to poor adverse events, particularly with regard to postoperative wound healing and increased requirements for analgesics (Akutay & Ceyhan, 2023). Benzodiazepines are commonly prescribed to manage preoperative anxiety, but their increased use in older patients has been linked with adverse effects, including delirium, respiratory depression, delayed postoperative mobilization, and longer hospital stays (Gauthier et al., 1992; Inouye, 2006; Lader et al., 2009). Because of the disadvantages associated with preoperative analgesics and depressants, there is growing interest in adopting multimodal approaches for perioperative pain and anxiety for enhanced surgical recoveries (Fekrat et al., 2006; Gürsoy et al., 2016; Kagan & Bar‐Tal, 2008; Mitchell, 2010).

Some alternative modalities for managing perioperative pain and anxiety are meditation and mindfulness‐based therapies (Hilton et al., 2016). Meditation can be defined as a form of mental training that seeks to alleviate stressful responses by improving an individual's psychological well‐being (Sampaio et al., 2017; Tang et al., 2015). There are many different forms of meditation that have been shown to improve patient outcomes across all domains, most of which incorporate mindfulness‐based practices (Hymowitz et al., 2024; Packiasabapathy et al., 2019; Raffone et al., 2019; Reynolds & Jahromi, 2022). Several reviews and clinical trials have published the effects of meditation on pain in recent years, particularly with respect to chronic conditions such as back pain, labor pain, and neuropathic pain (Cherkin et al., 2016; Hilton et al., 2016; Hussain & Said, 2019). However, the effects of meditation on acute preoperative and postoperative outcomes have not been adequately summarized.

Thus, the purpose of this study was to conduct a systematic review of randomized controlled trials (RCTs) evaluating the efficacy of meditation practices on pain and anxiety for individuals undergoing in‐hospital procedures or surgeries. To our knowledge, our study will be the first systematic review to assess the efficacy of meditative practices for pain and anxiety in the perioperative setting.

## 2 METHODS

### 2.1 Search strategy

This systematic review was conducted following the Preferred Reporting Items for Systematic Reviews and Meta‐Analyses (PRISMA) guidelines (Moher et al., 2009). The medical librarian searched electronic literature databases. Search concepts included “Surgical Procedures,” “Mindfulness,” and “Pre‐Peri‐ or Postoperative.” Search strategies were formulated using a combination of standardized index terms and keywords. The last updated search was conducted up for articles from inception to May 2024. Searches were run in PubMed, MEDLINE and adapted for Embase, PsycINFO, APA PsycINFO, All EBM Reviews Scopus, and Web of Science. Animal studies were excluded.

### 2.2 Study selection

Eligible studies involved (1) adult patients (≥18 years) undergoing any invasive procedures (surgery, biopsy, and grafting); (2) evaluation of mindfulness, meditation, and/or yoga practices for treatment of preoperative pain and/or anxiety; (3) comparisons of the intervention with another meditation practice or usual care; (4) reports of short‐term pre‐ and postoperative measurements of pain and anxiety (±90 days) via a standardized scoring system; (5) publication in English. Only full texts of RCTs were included. Non‐invasive treatments, single‐group studies, studies with no measurement of pain or anxiety, and interventions that did not involve mindfulness‐based practices were excluded. Two independent reviewers screened the titles and abstracts of all retrieved citations. Furthermore, reviews,meta‐analyses, case reports, and letter to the editors were excluded. Citations that were potentially eligible by either or both reviewers were then assessed via full‐text screening. This process was documented via an electronic database and the reasons for exclusions were recorded. Reviewers then worked independently to extract study outcomes and characteristics using a standardized extraction form. An additional reviewer examined the extraction and resolved conflicts. The risk of bias of the included RCTs was evaluated with the Cochrane Collaboration's risk of bias 2 tool (Higgins et al., 2011).

### 2.3 Outcome measures

The following data were collected: study characteristics (author and surgical procedure), demographics (age and number of females), and type of meditative practice employed. The primary outcomes included baseline and postoperative pain scores, baseline and postoperative anxiety scores, and time points for baseline and follow‐up assessments. Details of the interventions reported for each study are included in Table 1.

**TABLE 1:** Baseline study characteristics.

Author (year)	Country	Surgical procedure	Sample size (intervention: control)	Number of females (%)	Mean age, years (SD)	Intervention(s)	Control
Coelho (2018)	Brazil	Breast biopsy	82 (41:41)	82 (100%)	44.3 (± 13.6)	Mindfulness‐based Meditation: 1 session pre‐op	Standard care
Ratcliff (2019)	USA	Breast biopsy	84 (34:33:17) GM:34 FB: 33 C: 17	84 (100%)	55.4 (±11.3)	1. Guided mindfulness‐based mediation (GM): single 10 min session pre‐op 2. Focused breathing exercises (FB): single 10 min session pre‐op	Standard care: single 10 min neutral content audio
Soo (2016)	USA	Breast biopsy	121 (46:46:46)	121 (100%)	53.0 (±12.3)	1. Loving–kindness meditation (LKM): 3 × 20 min. recording via audio headphones pre‐op 2. Music therapy (MT): 3 × 20 min recording via audio headphones pre‐op	Supportive dialogue (SD): supportive dialogue from staff during biopsy
Wren (2019)	USA	Breast biopsy and breast cancer surgery	135 (45:46:44) LKM: 45 MT: 46 C: 44	135 (100%)	55.76 (±10.8)	1. Loving–kindness meditation (LKM): audio during biopsy, 20 min/day home practice, and 15 min booster conversation with staff 2. Music therapy (MT): music during procedure + 20 min music listening once per day	Supportive dialogue (SD): supportive dialogue from staff during biopsy
Rao (2017)	India	Breast cancer surgery	69 (33:36)	69 (100%)	49.1 (±9.45)	Integrated yoga program and didactics lectures: four 1 h sessions in pre‐ and post‐op periods, followed by 3 in person sessions every 6 weeks during adjuvant treatment every 21 days. 1 h home practice sessions 6 days/week	Supportive counseling (SC): conventional 1 h counseling session on treatment and side effects
Dion (2016)	USA	Breast Reconstruction	40 (20:20)	40 (100%)	47.7 (±8.4)	Massage and guided meditation: 3 sessions, each 20 min. During post‐op days: 1, 2, 3	Massage only: 3 sessions, each 20 min. During post‐op days: 1, 2, 3
Dowsey (2019)	Australia	Total hip or knee arthroplasty	127 (65:62)	92 (72.4%)	65.5 (±9.3)	Mindfulness‐based stress reduction (MBSR): 8‐session group‐based MBSR pre‐op, weekly 2.5 h sessions, a 7 h session held in week six, and a “booster” day‐long workshop 3 months post‐surgery	No intervention
Kiran (2017)	India	Coronary artery bypass graft	147 (73:74)	113 (76.9%)	55.4 (± 5.9)	Raja Yoga (RY): 3 sessions, 30 min per session	No intervention
Raghuram (2014)	India	Coronary artery bypass grafting	250 (129:121)	0 (0%)	52.9 (± 6.6)	Yoga lifestyle program: 30 min live sessions of Yoga and life counseling	Standard physiotherapy program (SPP): 30 min live physiotherapy sessions
Hou (2019)	China	Percutaneous coronary intervention	61 (30:31)	12 (19.7%)	58.7 (± 10.92)	Mindfulness‐based stress reduction (MBSR): 6‐week MBSR via audio, face to face, and booklet; 3 sessions total performed every 2 weeks for 30–40 min per session, post‐op	No intervention
Lisann‐Goldman (2019)	USA	Cardiac surgery with intra‐op biopsy	25 (12:13)	6 (24%)	64.1 (no SD)	Mindfulness with instructor followed by audio listening: 2 sessions per day during pre‐op and post‐op on days 1–5.	No intervention
Marinelli (2020)	Italy	Pancreatic cancer surgery	114 (65:49)	61 (53.5%)	62 (no SD)	Psychological intervention to improve mindfulness: single session pre‐op	No intervention
Beiranvand (2014)	Iran	C‐section	160 (80:80)	160 (100%)	27.5 (± 4.0)	Prayer and meditation via headphones listening (Muslim women): 1 session early pre‐op	No intervention
Haisley (2020)	USA	Foregut surgery	52 (26:26)	38 (73.1%)	63.5 (no SD)	VR meditation/mindfulness sessions: 6 sessions total, each lasting over 4 min, 3 sessions pre‐op, 3 sessions post‐op	No intervention
Agarwal (2012)	India	Procedure for prolapse and hemorrhoids (PPH)	100 (50:50)	18 (18%)	48.3 (no SD)	PPH with perioperative yoga exercises: yoga three times in a day for 10 min duration each and counseling for 10 days	PPH without perioperative yoga exercises
Lu (2022)	Taiwan	Laparoscopic cholecystectomy	66 (33:33)	39 (57.4%)	55.6 (± 11.7)	Guided imagery meditation: once before surgery, and then twice a day after surgery for 15–20 min at a time.	Usual care: oral guidance to patients regarding pain control techniques

## 3 RESULTS

**FIGURE 1:**
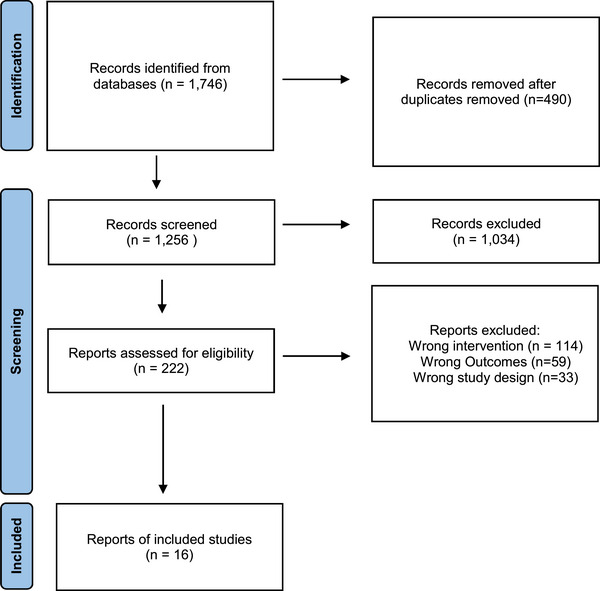
Preferred Reporting Items for Systematic Reviews and Meta‐Analyses flow diagram of inclusion and exclusion criteria.

The literature search identified 1746 citations, of which 1256 citations remained after removing duplicates. Full texts were reviewed for 222 citations and 16 RCTs met the inclusion criteria (Figure 1).

A total of 16 RCTs conducted between 2012 and 2022 were included in this systematic review. The included trials investigated the impact of different meditation practices on preoperative and postoperative pain and anxiety levels within a population that underwent different interventions including biopsies, grafting procedures, and surgeries. A total of 1633 patients were included in the study, with individual trials having sample sizes ranging from 25 to 250. The majority of the participants were female (65.5%, *n* = 1070) with seven studies involving only female participants. The mean age of the subjects was 54.3 (±9.9) years old. Breast‐related operations were the most common procedures (37.5%), followed by cardiac operations (25%), gastrointestinal (GI) procedures (25%), orthopedic operations (6.3%), and one C‐section (6.3%). Mindfulness‐based meditation practices were among the most prevalent interventions (43.8%), followed by yoga‐based meditation (31.3%). The frequency of the interventions ranged from a single in‐person session to multiple sessions scheduled within a 3‐month period that included both in‐person and home‐based exercises. Additional characteristics of the 16 included studies are summarized in Table 1.

### 3.1 Postoperative pain scores

Eight trials reported outcomes related to pain. Pain outcomes were assessed using standardized scoring systems, including the Visual Analog Scale (VAS) and Brief Pain Inventory (BPI). Five of the eight RCTs reported favorable outcomes on postoperative pain after various meditation interventions. Two studies practiced loving–kindness meditation, one study involved prayer meditation, one study emphasized mindfulness training, and one study incorporated yoga practices (Agarwal et al., 2012; Beiranvand et al., 2014; Marinelli et al., 2020; Soo et al., 2016; Wren et al., 2019). A summary of these meditative practices is provided in Table 2. Two RCTs that had provided mindfulness‐guided meditation and one RCT that had practiced virtual reality meditation with mindfulness sessions did not show a significant difference in postoperative pain scores compared to control. However, all other interventions did reveal a significant reduction in pain scores post‐op (Coelho et al., 2018; Haisley et al., 2020; Ratcliff et al., 2019).

**TABLE 2:** Pain scores and outcomes.

Author (year of publication)	Surgical interventions	Experimental intervention	Intervention pain scale and score	Control pain scale and score	Conclusion
Coelho (2018)	Breast biopsy	Mindfulness‐based meditation	Final VAS: 1.30 ± 1.10	Final VAS: 1.50 ± 1.20	Mindfulness‐based meditation did not show a significant difference in post‐op pain compared to control
Ratcliff (2019)	Breast biopsy	1. Guided mindfulness‐based mediation (GM) 2. Focused breathing exercises (FB)	1. Baseline VAS: 0.86 ± 1.65 Final VAS: N/A 2. Baseline VAS: 1.07 ± 1.55 Final VAS: N/A	1. Baseline VAS: 0.94 ± 1.53 Final VAS: N/A 2. Data N/A	Guided mindfulness‐based training pre and post biopsy did not show significant differences in pain scores
Soo (2016)	Breast biopsy	1. Loving–kindness meditation (LKM) 2. Music therapy (MT)	1. VAS: 0.84 ± 0.41 Final VAS: 0.73 ± 0.44 2. VAS: 0.59 ± 0.37 Final VAS: 1.74 ± 0.40	VAS: 0.38 ± 0.35 Final VAS: 1.50 ± 0.38	Patients undergoing LKM had significantly less postoperative pain when compared to standard care. There were no difference in pain between MT and standard care
Wren (2019)	Breast biopsy and breast cancer surgery	1. Loving–kindness meditation (LKM) 2. Music therapy (MT)	1. BPI: 1.81 ± 2.05 Final BPI: 1.58 ± 1.42 2. BPI: 1.87 ± 2.56 Final BPI: 1.18 ± 1.42	BPI: 1.29 ± 1.44 Final BPI: 0.70 ± 0.84	LKM and MT had significantly smaller reports of body pain compared to control
Marinelli (2020)	Pancreatic cancer surgery	Single session psychological intervention to improve mindfulness	Final BPI (physical): 4.3 ± 1.6 Final BPI (emotional): 2.8 ± 1.8	Final BPI (physical): 4.8 ± 2.3 Final BPI (emotional): 3.9 ± 2.4	Single session psychological intervention showed significant post‐op improvements in the emotional component of pain at 3 days
Beiranvand (2014)	C‐section	Prayer and meditation via headphones listening (Muslim women)	Baseline VAS: 2.7 Final VAS: 1.1	Baseline VAS: 2.4 Final VAS: 3	Meditation significantly decreased postoperative pain at 3‐ and 6‐h marks
Haisley (2020)	Foregut surgery	VR meditation/mindfulness sessions	Baseline VAS: 1.7 Post‐op VAS: 3.8	Baseline VAS: 1.2 Post‐op VAS: 3.9	VR‐based meditation and mindfulness did not show significantly decreased post‐op pain scores compared to control
Agarwal (2012)	Procedure for prolapse and hemorrhoids (PPH)	PPH with perioperative yoga exercises	Baseline # of patients > 5 point VAS: 11 Final # of patients > 5 point VAS: 0	Baseline # of patients > 5 point VAS: 34 Final # of patients > 5 point VAS: 5	Yoga was significantly associated with improvement in post‐op pain

Abbreviations: BPI, Brief Pain Inventory scale; VAS, Visual Analog Scale.

### 3.2 Postoperative anxiety levels

Anxiety outcomes were analyzed using the Depression Anxiety Stress Scale (DASS), State‐Trait Anxiety Inventory (STAI), the Hospital Anxiety and Depression Scale (HADS), and the Back Anxiety Inventory (BAI). Only the anxiety and anxiety‐state subgroups were reported for the HADS and STAI scales, respectively. Ten of the 11 RCTs reported reductions in anxiety outcomes, with five of these studies incorporating mindfulness‐based meditation interventions (Coelho et al., 2018; Hou et al., 2019; Kiran et al., 2017; Lisann‐Goldman et al., 2019; Lu et al., 2022; Marinelli et al., 2020; Raghuram et al., 2014; Rao et al., 2017; Ratcliff et al., 2019; Wren et al., 2019). Five RCTs had incorporated mindfulness‐guided meditation, three had provided yoga meditation, one RCT had emphasized loving–kindness meditation, and one implemented guided imagery meditation. A summary is provided in Table 3. The remaining outcomes that reduced anxiety were derived from consistent yoga and loving–kindness practices. The final RCT, which introduced massages paired with guided meditation, reported no significant differences in pre‐ and postoperative anxiety scores (Dion et al., 2016).

**TABLE 3:** Anxiety scores and outcomes.

Author (year of publication)	Surgical interventions	Experimental intervention	Intervention anxiety scale and score	Control anxiety scale and score	Conclusion
Coelho (2018)	Breast Biopsy	Mindfulness‐based meditation	Final DASS anxiety: 6.9 ± 6.9	Final DASS Anxiety: 9.4 ± 8.2	Mindfulness‐based meditation was not significantly associated with reduced levels of perceived anxiety
Ratcliff (2019)	Breast biopsy	1. Guided mindfulness‐based mediation (GM) 2. Focused breathing exercises (FB)	1. Baseline VAS‐anxiety: 2.96 ± 2.69 Final VAS‐anxiety: N/A 2. Baseline VAS‐anxiety: 3.33 ± 2.67 Final VAS‐anxiety: N/A	Baseline VAS‐anxiety: 2.56 ± 2.99 Final VAS‐anxiety: N/A	Guided mindfulness training and focused breathing training did not show significant differences in perceived anxiety scores after group specific activities. However, women in the guided mindfulness group reported a greater reduction in anxiety scores during the breast biopsy
Wren (2019)	Breast biopsy and breast cancer surgery	1. Loving–kindness meditation (LKM) 2. Music therapy (MT)	1. Baseline STAI‐anxiety state: 41.72 ± 11.35 Final STAI‐anxiety state 37.80 ± 10.32 2. Baseline STAI‐anxiety state: 47.26 ± 17.35 Final STAI‐anxiety state: 33.18 ± 11.37	Baseline STAI‐anxiety state: 43.82 ± 12.3 Final STAI‐anxiety state: 38.81 ± 11.11	Loving–kindness and meditation showed significantly decreased anxiety levels in the follow‐up period compared to control. Adding music therapy did not cause a greater decrease in anxiety scores over time
Marinelli (2020)	Pancreatic cancer surgery	Single session psychological intervention to improve mindfulness	Baseline STAI‐anxiety state: 43.1 ± 13.7 Final STAI‐anxiety state: 28.2 ± no SD	Baseline STAI‐anxiety state: 43.4 ± 12.1 Final STAI‐anxiety state: NR	There was a significant decrease in state anxiety within the intervention group
Rao (2017)	Breast cancer surgery	Integrated yoga program, live session, and didactics lectures	Baseline STAI‐anxiety state: 44.20 ± 10.90 Final STAI‐anxiety state: 34.00 ± 3.90	Baseline STAI‐anxiety state: 49.6 ± 12.0 Final STAI‐anxiety state: 38.40 ± 8.10	The integrated yoga program significantly decreased perceived anxiety scores in the immediate post‐op period
Dion (2016)	Breast reconstruction	Massage and guided meditation	Baseline VAS‐anxiety: 3.42 Final VAS‐Anxiety: 1.58	Baseline VAS‐anxiety: 4.47 Final VAS‐Anxiety: 1.84	Adding massage to meditation did not influence pre‐ or post‐op perceived anxiety levels
Kiran (2017)	Coronary artery bypass graft	Raja Yoga (RY)	Baseline VAS‐anxiety: 7.10 ± 0.80 Final VAS‐anxiety: 0.69 ± 1.10	Baseline VAS‐anxiety: 6.87 ± 0.68 Final VAS‐anxiety: 5.6 ± 1.38	Raj Yoga training showed significant decreases in anxiety scores from post‐op days 2 and 3 compared to control
Raghuram (2014)	Coronary artery bypass grafting	Yoga lifestyle program	Baseline HADS‐anxiety: 7.42 ± 3.40 Final HADS‐anxiety: 5.75 ± 3.46	Baseline HADS‐anxiety:7.84 ± 3.05 Final HADS‐anxiety: 6.15 ± 2.98	Within the yoga group, there was a significantly decreased score between baseline and final follow‐up. However, the difference in anxiety scores was comparable between yoga and control
Hou (2019)	Percutaneous coronary intervention	Mindfulness‐based stress reduction (MBSR)	Baseline HADS‐anxiety: 15.67 ± 5.42 Final HADS‐anxiety: 10.50 ± 4.37	Baseline HADS‐anxiety: 16.94 ± 6.66 Final HADS‐anxiety: 14.74 ± 6.90	MBSR via audio, in‐person, and booklet significantly reduced perceived anxiety levels throughout the 6‐week intervention
Lisann‐Goldman (2019)	Cardiac surgery with intra‐op biopsy	Mindfulness with instructor followed by audio listening	Baseline HADS‐anxiety: 5 Final HADS‐anxiety: 2.5	Baseline HADS‐anxiety: 3 Final HADS‐anxiety: 3	Mindfulness training in person followed by audio recording was comparable to control in reducing anxiety levels
Lu (2022)	Laparoscopic cholecystectomy	Guided imagery meditation (GIM)	Baseline BAI: 6.00 ± 3.40 Final BAI: 0.42 ± 0.97	Baseline BAI: 8.55 ± 6.58 Final BAI: 4.79 ± 7.55	The results showed that GIM can significantly improve the postoperative quality of life.

Abbreviations: BAI, Beck Anxiety Inventory; DASS, Depression, Anxiety, and Stress Scale; HADS, Hospital Anxiety and Depression Scale (HADS‐Anxiety subgroup reported); STAI, State‐Trait Anxiety Inventory (STAI‐anxiety state subgroup reported); VAS, Visual Analog Scale.

### 3.3 Quality of studies

Regarding the quality of the studies included in this systematic review, the RCTs were not blinded due to the nature of the interventions, possibly introducing performance bias when analyzing the primary outcomes. Since the majority of the studies compared a particular meditation practice to a normal standard of care, patients allocated to the intervention groups could be more biased in reporting their outcomes since they knew they were undergoing a meditation practice for pain or anxiety reduction. Second, the primary outcomes were based on patient‐reported scoring systems. This makes the outcomes subjective and difficult to generalize without large sample sizes. Additionally, due to the heterogeneity across the different RCTs and the different outcome measures a meta‐analysis of the results could not be conducted.

To assess the risk of bias, version 2 of the Cochrane Collaboration risk of bias tool (RoB2) was utilized for this systematic review. A total of 9 of the 16 trials had a high risk of bias within at least one category (Table 4; Higgins et al., 2011). The most common biases were related to deviations from intended interventions and biases in the measurement of outcomes.

**TABLE 4:** Assessment of the quality of included studies according to the Cochrane risk of bias tool for randomized trials.

Author, year	Bias arising from the randomization process	Bias due to deviations from intended interventions	Bias due to missing outcome data	Bias in measurement of the outcome	Bias in selection of the reported result
Coelho, 2018 (Coelho et al., 2018)	+	+	+	?	+
Ratcliff, 2019 (Ratcliff et al., 2019)	+	?	+	−	+
Soo, 2016 (Soo et al., 2016)	+	?	+	−	?
Wren, 2019 (Wren et al., 2019)	+	?	?	−	?
Rao, 2017 (Rao et al., 2017)	+	?	+	+	?
Dion, 2016 (Dion et al., 2016)	?	−	+	+	+
Dowsey, 2019 (Dowsey et al., 2014)	+	?	+	?	+
Kiran, 2017 (Kiran et al., 2017)	?	?	+	−	+
Raghuram, 2014 (Raghuram et al., 2014)	?	?	+	+	+
Hou, 2019 (Hou et al., 2019)	?	−	+	+	+
Lisann‐Goldman, 2019 (Lisann‐Goldman et al., 2019)	+	?	+	+	+
Marinelli, 2020 (Marinelli et al., 2020)	−	−	?	?	+
Beiranvand, 2020 (Beiranvand et al., 2014)	−	?	+	−	+
Haisley, 2020 (Haisley et al., 2020)	?	−	?	+	?
Agarwal, 2012 (Agarwal et al., 2012)	+	?	?	+	?
Lu, 2022 (Lu et al., 2022)	+	?	?	+	+

## 4 DISCUSSION

The results of this review present evidence that meditation can provide short‐term, postoperative pain and anxiety relief for various procedures and surgeries. Yoga and loving–kindness meditation proved to be effective in dampening postoperative pain and anxiety levels when compared to preoperative levels (Agarwal et al., 2012; Dowsey et al., 2014; Kiran et al., 2017; Raghuram et al., 2014; Rao et al., 2017; Soo et al., 2016; Wren et al., 2019). Mindfulness‐based meditations appeared to be less effective for postoperative pain levels; however, these meditations were effective for alleviating postoperative anxiety levels, with five studies delineating lower postoperative anxiety scores (Coelho et al., 2018; Haisley et al., 2020; Hou et al., 2019; Lisann‐Goldman et al., 2019; Marinelli et al., 2020; Ratcliff et al., 2019). Although there are only a few studies present in this systematic review, the results from these randomized trials seem to indicate immediate benefits to incorporating yoga, loving–kindness, and mindfulness‐based meditations for preoperative anxiety‐related outcomes.

There is a growing body of evidence that supports the benefits of meditation for different types of pain and anxiety within different populations, such as people diagnosed with insomnia, dementia, generalized anxiety disorder, and arterial hypertension (Ponte Márquez et al., 2019). Recently, a previous systematic review and meta‐analysis by Hilton et al. (2016) delineated improvements in pain and depressive symptoms for adults who underwent mindfulness meditation practices for the treatment of chronic pain. The analysis by Hilton et al. revealed that meditations of various lengths were correlated with statistically significant improvement in overall pain, depression, and overall mental quality of life scores with minimal adverse events. They also analyzed two studies that showed decreased levels of analgesic use by patients who underwent meditation; however, the results from this particular analysis were varied (Ayers et al., 2004). Our systematic review has suggested that meditation can be beneficial in perioperative settings. However, the heterogeneity in the meditation practices employed, the duration of each treatment, and the surgical interventions assessed made it difficult to generalize any conclusion about a particular population or specialty.

Mindfulness‐based meditation was assessed across six RCTs for pain and anxiety (Coelho et al., 2018; Haisley et al., 2020; Hou et al., 2019; Lisann‐Goldman et al., 2019; Marinelli et al., 2020; Ratcliff et al., 2019). Coelho et al. (2018) and Ratcliff et al. (2019) assessed the effects of mindfulness‐based meditation on women undergoing breast biopsies and found no significant group difference in pain and anxiety scores between their intervention and control groups. Coelho et al. (2018) suggested that pain is not typically a major issue during breast biopsies which may explain why no group differences were reported. Furthermore, Ratcliff et al. (2019) indicated that since most of the women received local anesthesia before their biopsies and had preoperative pain scores of 0, there was not enough evidence to suggest whether mindfulness‐based meditation actually had any impact on pain. Haisley et al. (2020) assessed the role usage of VR‐directed mindfulness meditation before minimally invasive foregut surgery and reported comparable postoperative differences in pain and anxiety between the intervention and control. The authors elaborated on their findings, suggesting that patients who had undergone more complex surgeries and those who were uncomfortable with the technology due to digital naivety because of older age, tended to experience no difference in pain or anxiety within their intervention (Haisley et al., 2020). On the other hand, a couple of studies did find significant differences in pain and anxiety after mindfulness‐based meditation. Marinelli et al. (2020) assessed the effects of a one‐session psychological mindfulness‐based intervention in patients with pancreatic cancer about to undergo a major procedure and reported significant reductions in patient‐reported anxiety and pain scores when compared to a control group. Their results suggested that this form of psychological intervention was most efficacious right before surgery, suggesting that specific time frames could be important predictors of success (Marinelli et al., 2020). Hou et al. (2019) employed a one‐on‐one telephone‐adapted mindfulness‐based stress reduction program on patients undergoing percutaneous coronary interventions and found significantly greater reductions in HADS scores when compared to control groups. The authors suggested that patients who engaged in a mindfulness intervention could potentially alter psychological symptoms and perceived stress levels through the enhancement of mindfulness (Hou et al., 2019).

Five RCTs assessed the impact of other forms of meditation on pain and anxiety (Beiranvand et al., 2014; Dion et al., 2016; Lu et al., 2022; Soo et al., 2016; Wren et al., 2019). Dion et al. (2016) explored the postoperative anxiety effects of massage therapy in combination with meditation for breast cancer patients who had undergone autologous tissue reconstruction. No difference was found between the patients who received the massage alone versus those who received the massage with meditation, suggesting that patients were not alert and in the correct psychological state to take on a demanding mental task due to the influence of postoperative pain medications most patients were on (Dion et al., 2016). Soo et al. (2016) compared the effects of music therapy and loving–kindness meditation to standard care for women about to undergo an image‐guided breast biopsy. Both the meditation and music groups had reported significantly greater pain reduction scores when compared to the standard care, indicating that music and loving–kindness meditation are capable of enhancing patient experiences prior to breast biopsies (Soo et al., 2016). Beiranvand et al. (2014) studied the effect of prayer meditation on Muslim women who were about to undergo a cesarean surgery and found significant improvements in pain scores 3 and 6 h postoperatively when compared to a control group. The authors suggested that Muslim women tend to practice prayer meditation on a routine basis and that prayer before their operation allowed them to feel more comfortable and at peace during the surgery (Beiranvand et al., 2014). Lu et al. (2022) recently studied the impact of guided imagery meditation on postoperative anxiety in patients who had laparoscopic cholecystectomy surgery. The majority of the patients had reported postoperative anxiety related to concerns about abdominal incision tears and potential complications leading to prolonged hospitalization and increased medical expenses. The results showed significant improvements in anxiety scores for patients who had received guided imagery meditation on a compact disc (CD), suggesting self‐control and breathing exercises can help reduce anxiety and improve sleep quality (Lu et al., 2022).

Four RCTs assessed the impact of yoga on pain and anxiety (Agarwal et al., 2012; Kiran et al., 2017; Raghuram et al., 2014; Rao et al., 2017). Only one study reported findings related to pain (Agarwal et al., 2012). Agarwal et al. (2012) explored the impact of yoga on postoperative pain following the Procedure for Prolapse and Hemorrhoids (PPH) and found patients who had partaken in yogic exercises that stretched the muscles and connective tissue of the anorectal region had significantly improved pain scores when compared to control. Other studies found mixed results of yoga when it came to anxiety. Raghuram et al. (2014) compared the long‐term effects of yoga on patients who were enrolled in cardiac rehabilitation following coronary artery bypass surgery and found comparable differences in postoperative anxiety scores when compared to control. However, they reported significant differences in the baseline and final follow‐up scores within the yoga group itself. The authors suggested that yoga most likely reduced the stress arousal within the yoga group which may have decreased the pathogenesis for atherosclerosis and other cardiac complications associated with chronic stress (Raghuram et al., 2014). A similar study by Kiran et al. (2017) explored the role of Rajyoga meditation for modulating postoperative anxiety for patients about to receive a coronary artery bypass and found significantly improved anxiety levels on the second and fifth postoperative days. The authors found that the patients who practiced Rajyoga meditation had fewer increases in blood cortisol levels on the second and fifth postoperative days, suggesting that this form of yoga can help control postoperative anxiety levels (Kiran et al., 2017). Rao et al. (2017) assessed the impact of yoga on patients who underwent surgery for breast cancer and significant improvements in preoperative anxiety scores in those who had a yoga regimen versus control. These results suggested that yoga helped improve mood states and put patients in a relaxed state before going into the operating room (Rao et al., 2017).

Meditation's impact on behavior has been illustrated in multiple neuroanatomy studies that have hypothesized different brain regions to be responsible for inducing positive meditative effects (Hasenkamp et al., 2012; Hoge et al., 2013; Manna et al., 2010; Pagnoni, 2012). For instance, one meta‐analysis revealed that the caudate, entorhinal cortex, and medial prefrontal cortex elucidated higher levels of activity via functional magnetic resonance imaging (fMRI) and positron emission tomography (PET) scans when in a state of meditation (Sperduti et al., 2012). Other studies have investigated alterations in brain morphology in response to meditation (Grant et al., 2011; Hölzel et al., 2008; Lazar et al., 2005; Pagnoni & Cekic, 2007; Vestergaard‐Poulsen et al., 2009). One meta‐analysis utilized an activation‐likelihood estimation to suggest that eight regions in the brain were consistently morphed after prolonged periods of meditation, including the frontopolar cortex, the sensory cortices and insula, the hippocampus, and the anterior cingulate cortex (Fox et al., 2014).

There has been a growing interest in supplementing current preoperative practices with meditation to reduce opiate prescriptions for pain. Exogenous opioids are thought to deplete endogenous opioid production which may lead patients to take higher doses of these substances for longer periods (Corder et al., 2018). The long‐term effects of exogenous opioid use have been associated with higher rates of morbidity and mortality, inducing respiratory depressive effects when combined with other medications (Sen et al., 2016). In 2014, the American College of Occupational and Environmental Medicine's Evidence‐based Practice Guidelines demonstrated that opioids are not statistically more effective in the reduction of pain when compared to NSAIDs or other medications, for some indications (Hegmann et al., 2014). These findings, coupled with the contemporary opioid crisis in the United States, provide insight into why non‐analgesic alternatives should be applied when possible (Volkow & Blanco, 2021).

While our review indicates the potential benefits of meditation on perioperative pain and anxiety, it is crucial to acknowledge the significant heterogeneity among the included trials. Variations in the type of meditation interventions, the surgical cohorts, and the methodologies used across studies complicate the generalizability of our findings. Additionally, the inherent limitations of blinding in these studies present a high risk of performance bias. Patients and study team members were aware of the interventions, which may have influenced the reported outcomes. Furthermore, over 50% of the included studies were assessed as having a high risk of bias, primarily due to deviations from intended interventions and biases in outcome measurement. These factors underscore the need for standardized protocols and more rigorously designed studies to accurately assess the efficacy of meditation in this context.

Future research should focus on addressing the gaps and limitations identified in this review. Studies with larger sample sizes and more diverse populations are needed to enhance the generalizability of the results. Additionally, exploring the long‐term effects of meditation on postoperative recovery and the potential for reducing opioid consumption can provide valuable insights. Investigating the optimal timing, frequency, and types of meditation practices that yield the most significant benefits for specific surgical procedures will also be beneficial. Finally, incorporating objective measures, such as physiological markers of stress and pain, alongside patient‐reported outcomes, can help validate the efficacy of meditation interventions and minimize bias.

Although our results may signify promising postoperative outcomes of meditative interventions, there are limitations. First, our study design focused primarily on invasive, perioperative surgical procedures. It is plausible that pain and anxiety levels in patients undergoing non‐invasive procedures such as injections or ablations can exist and the impact of meditation on these settings should be analyzed. Second, our inclusion criteria focused on extracting data exclusively from published peer‐reviewed journals. Thus, it was difficult to formulate robust conclusions regarding the general population of patients undergoing invasive surgical procedures. Finally, as described earlier, there is heterogeneity in the types of surgical procedures and meditation interventions assessed, making it difficult to generalize any outcomes for a specific population. Further randomized trials are needed within each specialty to accurately measure the power of meditation in alleviating perioperative pain and anxiety.

## 5 CONCLUSION

This study has shown that there is a potential for incorporating meditation‐based interventions to alleviate pain and anxiety in patients before and after surgical procedures. Pairing meditation practices with the current standard of care can be an effective approach in reducing the use of analgesics and their concomitant side effects, while also improving patient‐reported outcomes.

## AUTHOR CONTRIBUTIONS

**Rami Rajjoub**: Conceptualization; investigation; methodology; software; formal analysis; writing—original draft; data curation. **Sally El Sammak**: Conceptualization; investigation; methodology; software; formal analysis; data curation; writing—original draft. **Tamim Rajjo**: Conceptualization; investigation; validation; writing—original draft; writing—review and editing; software. **Noora S. Rajjoub**: Conceptualization; investigation; writing—original draft; data curation. **Bashar Hasan**: Conceptualization; investigation; writing—original draft; writing—review and editing. **Samer Saadi**: Investigation; writing—original draft; writing—review and editing. **Adel Kanaan**: Writing—original draft; writing—review and editing; investigation. **Mohamad Bydon**: Investigation; writing—review and editing; supervision; project administration; resources.

## CONFLICT OF INTEREST STATEMENT

The authors declare no conflicts of interest.

## FUNDING

None

### PEER REVIEW

The peer review history for this article is available at https://publons.com/publon/10.1002/brb3.3640

## Data Availability

The data that support the findings of this study are available from the corresponding author upon reasonable request.
